# Supplementary material for: Cryo-EM captures early intermediate steps in dynein activation by LIS1
Source: Nat Commun. 2025 Aug 1;16:7054. doi: 10.1038/s41467-025-62185-z (PMC12317079; doi:10.1038/s41467-025-62185-z)
Supplement: Supplementary file 1 — Supplementary Information [file 41467_2025_62185_MOESM1_ESM.pdf]

SUPPLEMENTARY FIGURES

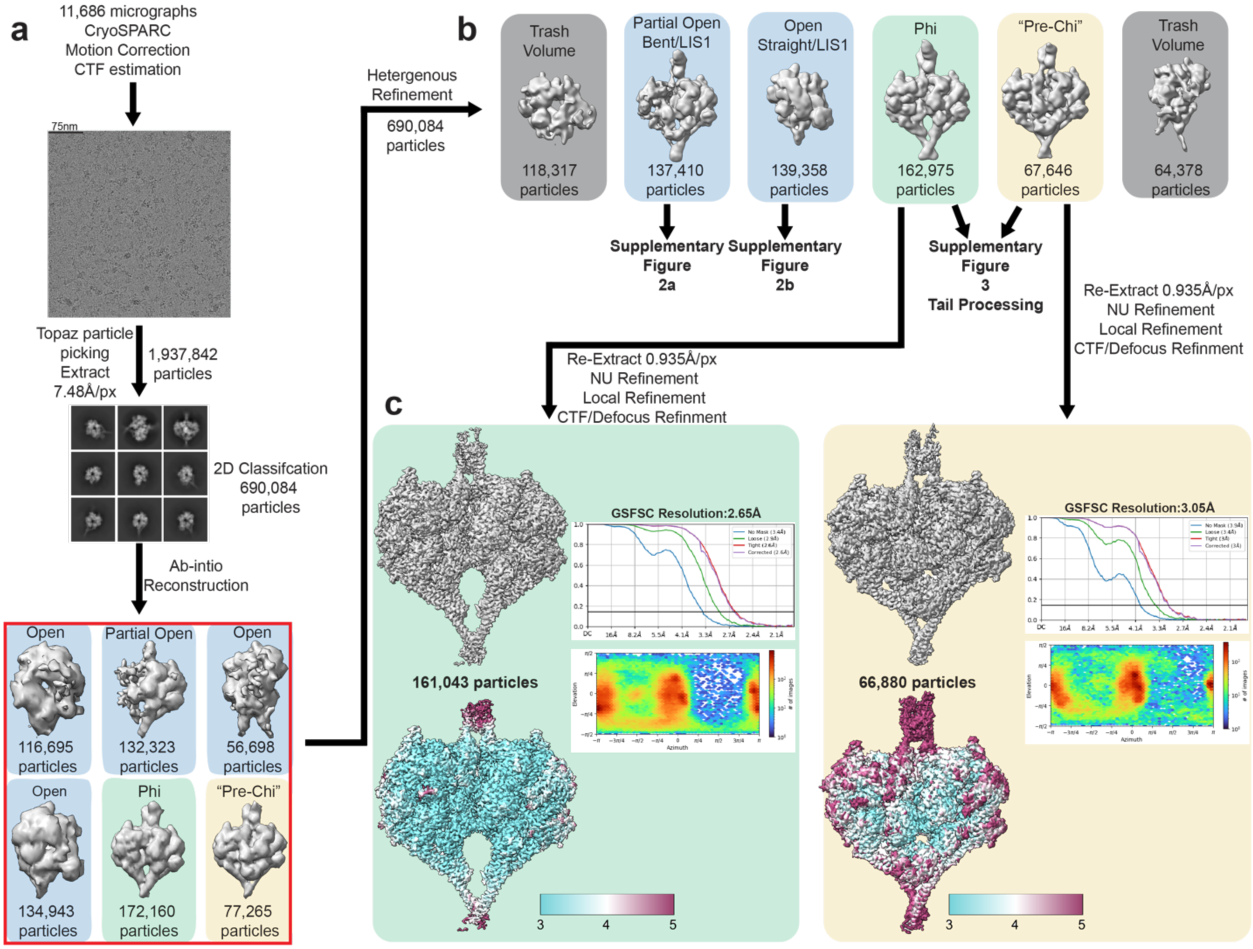

Supplementary Figure 1. Cryo-EM data processing workflow for human full-length dynein + LIS1 dataset

a. Dose-weighted movies were aligned, and CTF was estimated in cryoSPARC live. Particle extraction (binned by 8) was performed in cryoSPARC using a Topaz-trained model. Good particles from 2D classification jobs were used to generate six classes of ab-initio reconstructions in cryoSPARC. Ab-initio classes generated were color-coded according to the three main classes: Phi (light green), Pre-Chi (light yellow), and Open (light blue). All six maps and particles from the ab-initio classes were picked and carried on into heterogeneous refinement. **b.** Six volumes from the heterogeneous refinement job. Discarded volumes are highlighted in dark grey. Open classes were further separated and refined as shown in Supplementary Figure 2. **c.** Phi and Pre-Chi were unbinned and further refined to their final maps. The Fourier Shell Correlation (FSC) plots are shown next to the respective final maps. An elevation plot describing the angular distribution of particles used in the final reconstruction is shown next to their respective final maps.

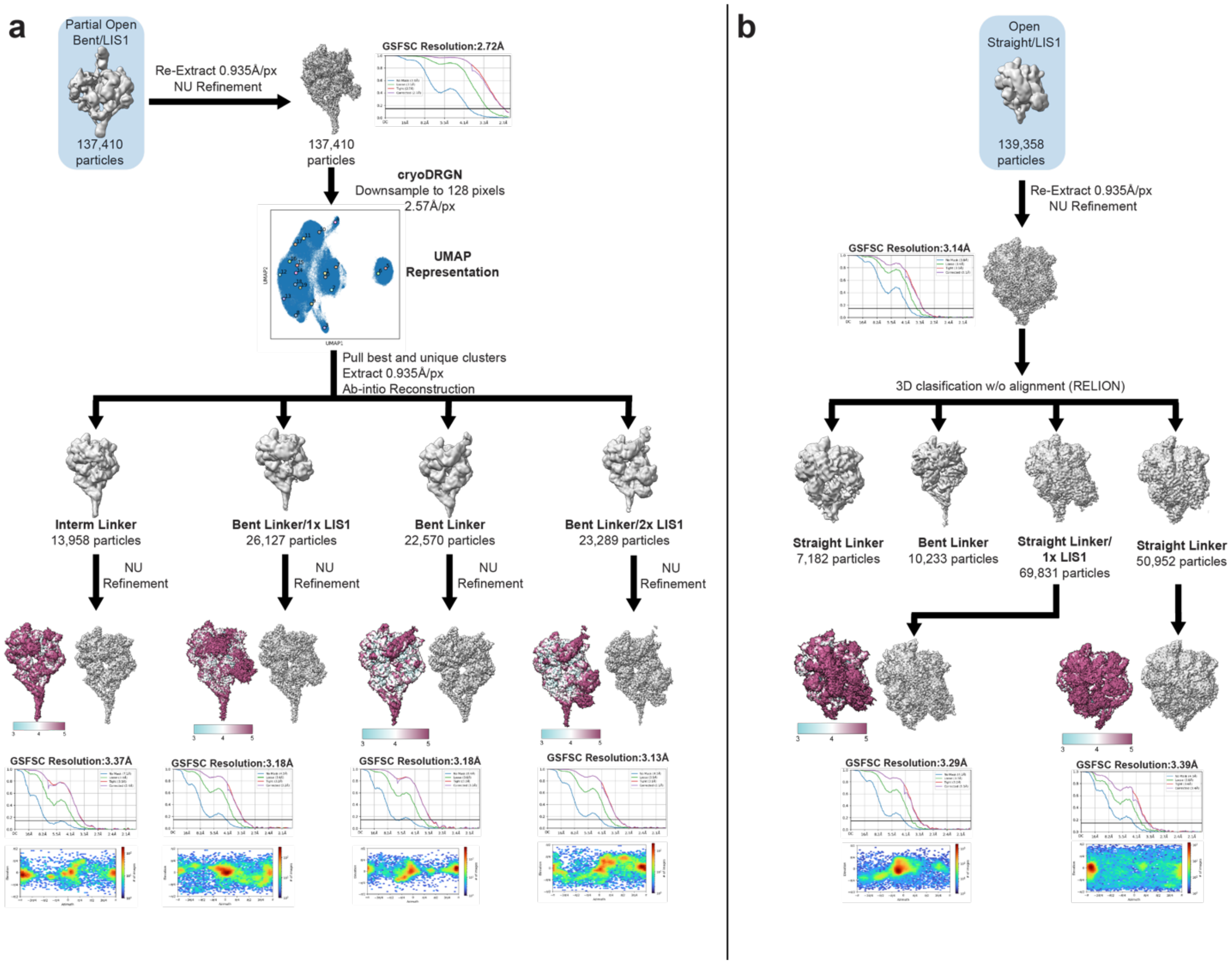

Supplementary Figure 2. Data processing workflow for Open species

a. Heterogeneous processing of Open species class with the linker in the bent conformation. The "Partial Open Bent/LIS1" class is the same one shown in Supplementary Figure 1. This class was unbinned and refined to obtain a map for cryoDRGN training. Particles were downsampled and subjected to a round of low-resolution cryoDRGN training. From the training, we pulled the best and most unique clusters from the training and returned the individual subclass particles into cryoSPARC. Each subclass is defined by their linker conformation and the presence or absence of LIS1. These subclasses were further refined to their final map. The Fourier Shell Correlation (FSC) plots are shown next to the respective final maps. **b**. Heterogeneous processing of the Open species class with the linker in the straight conformation. The "Open Straight/LIS1" class is the same one shown in Supplementary Figure 1b. This class was unbinned and refined to get a refined map for RELION 3D classification without alignment. The best subclasses were taken back into cryoSPARC to further refine their final map. The Fourier Shell Correlation (FSC) plots are shown next to the respective final maps. An elevation plot describing the angular distribution of particles used in the final reconstruction is shown next to their respective final maps.

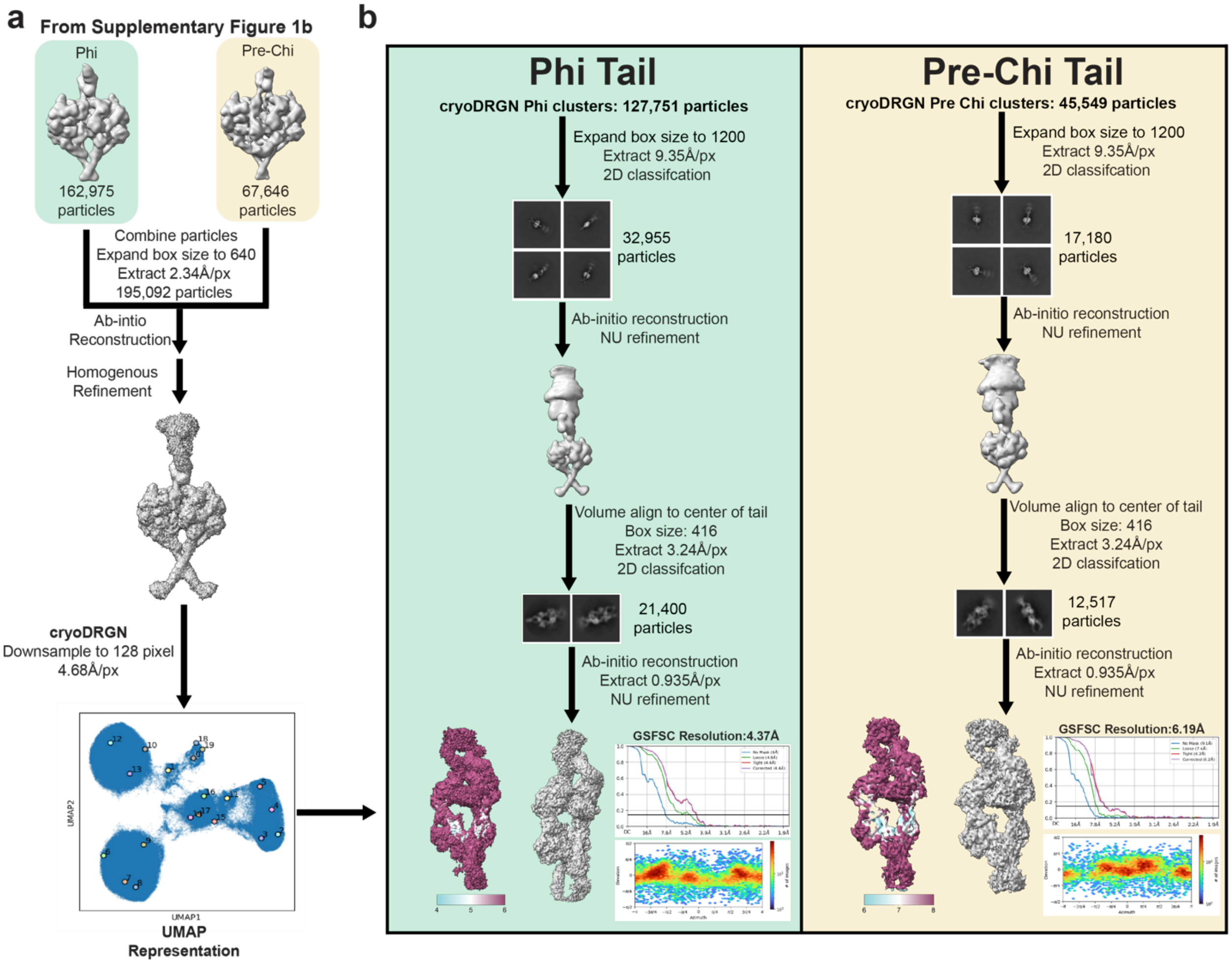

Supplementary Figure 3. Data processing of the tail domain of Phi and Pre-Chi

a. CryoDRGN analysis of combined Phi and Pre-Chi classes. The Phi and Pre-Chi classes are those shown in Supplementary Figure 1b. Particles from both classes were combined, and the box size was expanded further to include part of dynein's tail. A general refined consensus map was subjected to cryoDRGN low-resolution training, and Phi and Pre-Chi clusters were separated. **b**. Phi (green panel) and Pre-Chi (yellow panel) tail workflows. Both workflows use the same process, which is discussed in the methods section. The Fourier Shell Correlation (FSC) plots are shown next to the respective final maps. An elevation plot describing the angular distribution of particles used in the final reconstruction is shown next to their respective final maps.

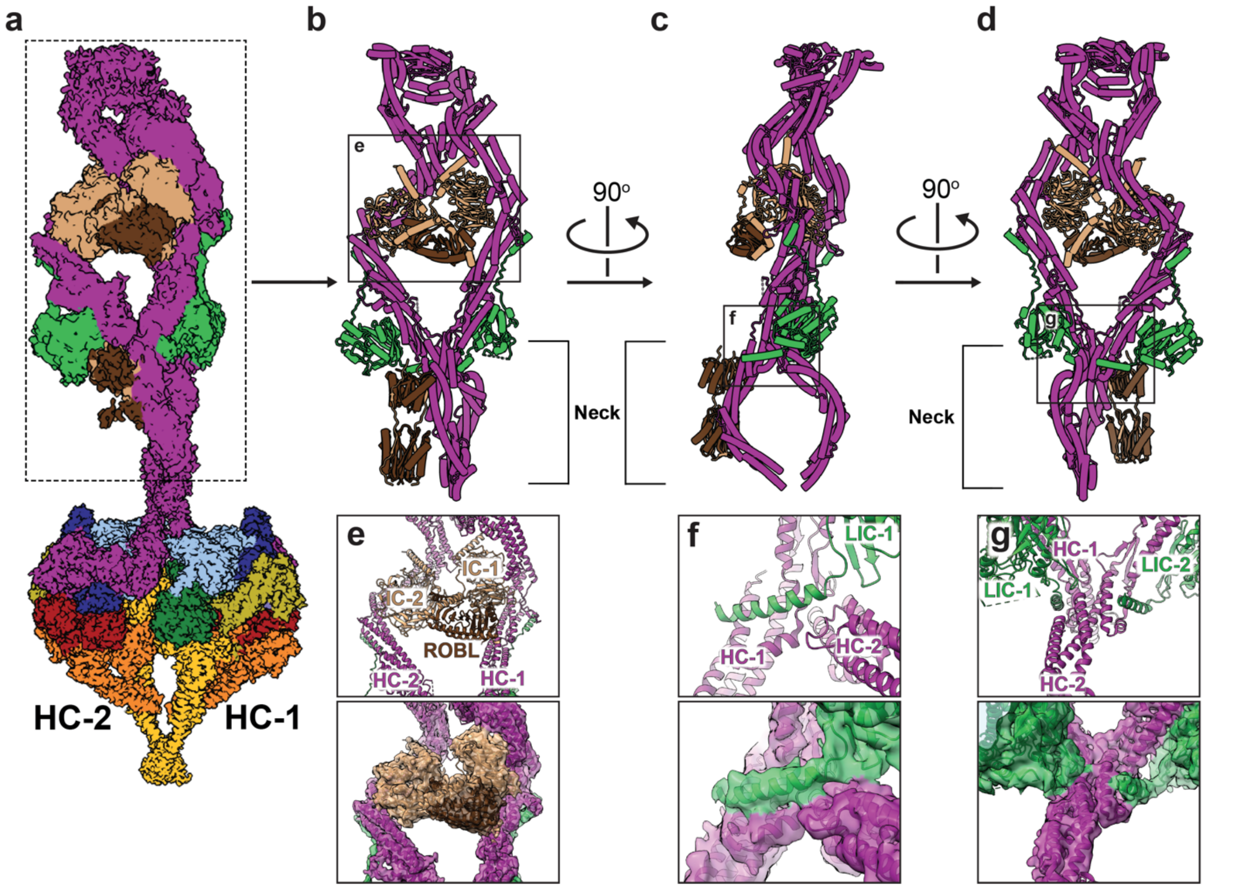

Supplementary Figure 4. Accessory chain interactions in Phi dynein.

a. Front view of the cryo-EM map of the motor and tail regions of Phi dynein. The heavy chain (HC) associated with each dynein monomer is highlighted for orientation. **b-d**. The model for the Phi dynein tail is shown in three orientations: (**b**) front view, (**c**) side view, and (**d**) back view. The neck region, which is the focus of panels (e) – (f) is highlighted. The black boxes indicate regions where our map revealed new interactions between accessory chains, and between them and dynein's heavy chain, with labels indicating the corresponding panels below (**e-g**). **e-g**. Close-up views of the three new interactions identified in our map, with the top panel displaying the model and the bottom panel showing the corresponding cryo-EM map, highlighting connecting densities. The numbering of the accessory chains reflects the heavy chain with which they are associated. **e**. Both intermediate chains (IC-1 and IC-2) interact with the light chain ROBL. **f**. Both light intermediate chains (LIC-1 and LIC-2) interact with their respective heavy chains (HC-1 and HC-2). **g**. LIC-1 interacts with both HC-1 and HC-2.

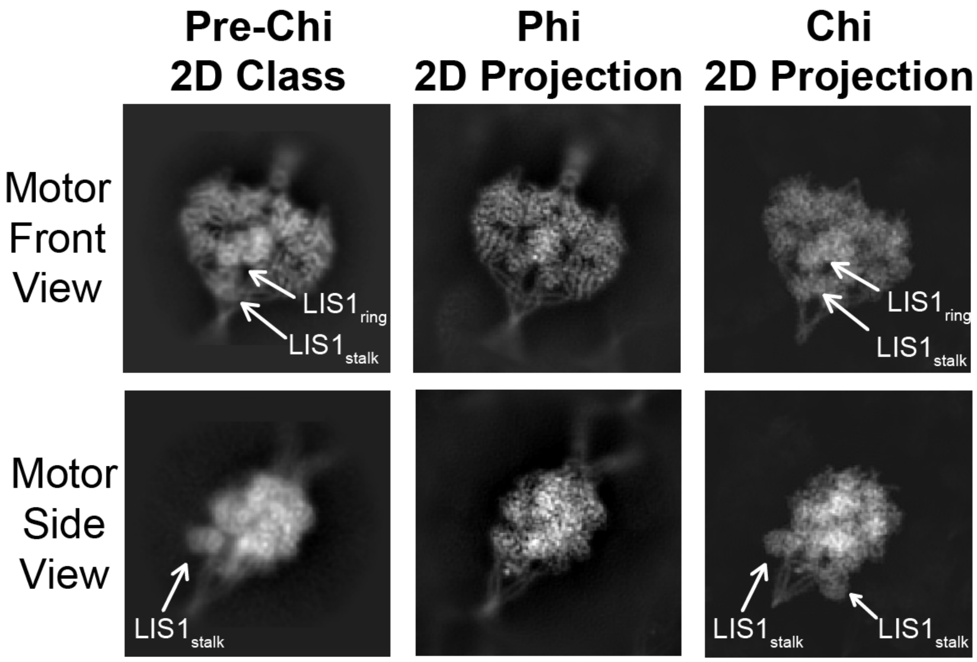

Supplementary Figure 5. Comparison of 2D class averages for Pre-Chi with previously known dynein models.

Projection matching between experimental class averages of Pre-Chi with projections from models of Phi and Chi.

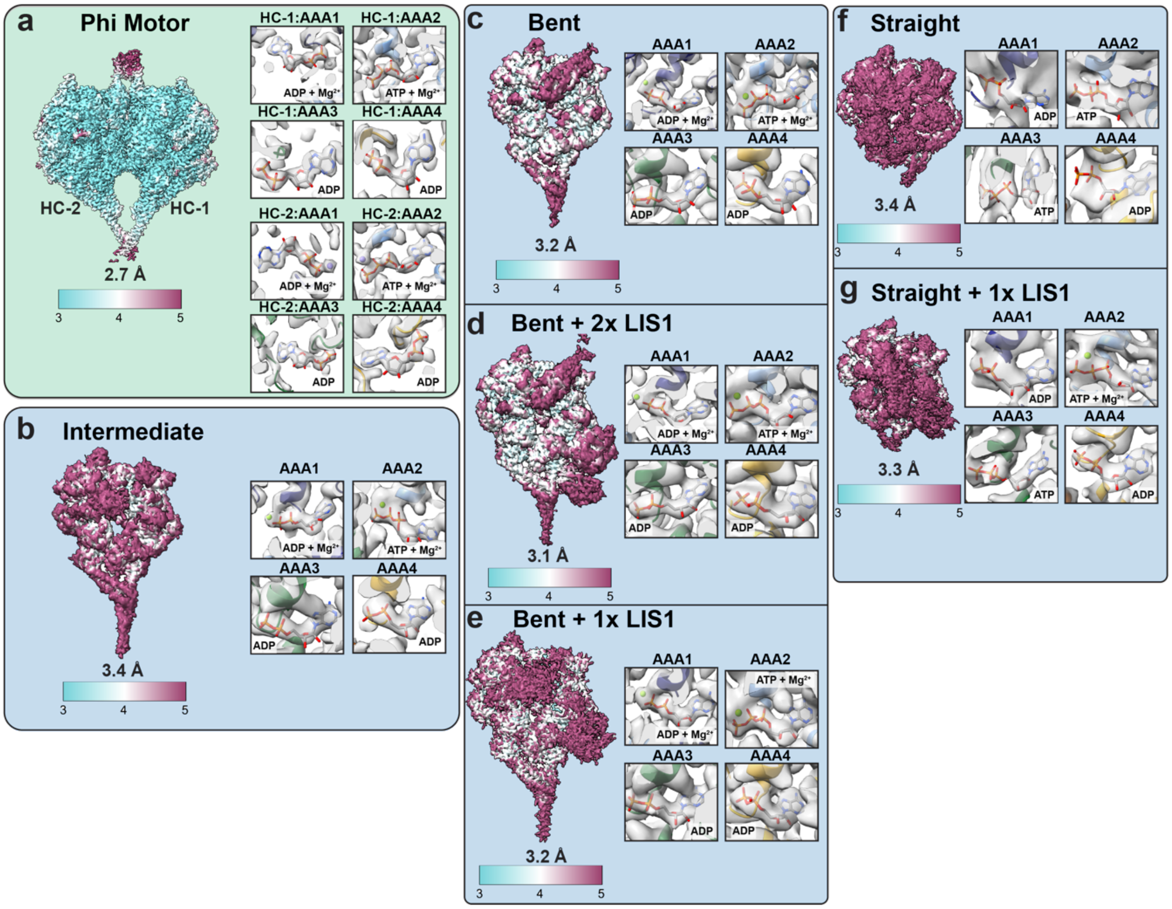

Supplementary Figure 6. Local resolution and nucleotide occupancies at AAA+ subunits.

Local resolution and views of the nucleotide-binding pockets for the indicated AAA+ modules for **a.** Phi motor, **b.** Intermediate, **c.** Bent, **d.** Bent + 2x LIS1, **e.** Bent + 1x LIS1**,** **f.** Straight, **g.** Straight + 1x LIS1.

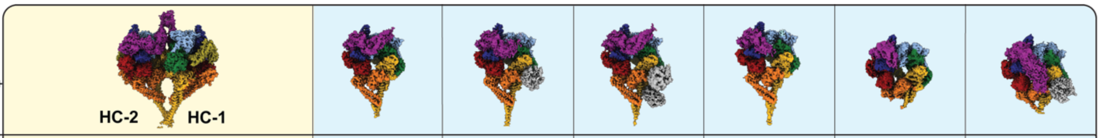

Legend: ● ATP; ATP + Mg^{2+}; ADP + Mg^{2+}; ○ ADP

Class	Phi motor		Bent	Bent+1xLIS1	Bent+2xLIS1	Interm.	Straight	Straight+2xLIS1
resol. (Å)	2.7		3.2	3.2	3.1	3.4	3.4	3.3
LIS1	0		0	1	2	0	0	1
Linker	HC-1 Bent	HC-2 Bent	Bent	Bent	Bent	Intermediate	Straight	Straight
Stalk	β	β	β	β	β	β	α	α
AAA1	ADP + Mg^{2+}	ADP + Mg^{2+}	ADP + Mg^{2+}	ADP + Mg^{2+}	ADP + Mg^{2+}	ADP + Mg^{2+}	ADP	ADP
AAA2	ATP + Mg^{2+}	ATP + Mg^{2+}	ATP + Mg^{2+}	ATP + Mg^{2+}	ATP + Mg^{2+}	ATP + Mg^{2+}	ATP	ATP + Mg^{2+}
AAA3	ADP	ADP	ADP	ADP	ADP	ADP	ATP	ATP
AAA4	ADP	ADP	ADP	ADP	ADP	ADP	ADP	ADP

Supplementary Figure 7. Conformations and nucleotide states of the Phi and Open motor domains.

The table summarizes the conformations and nucleotide states of the Phi (yellow background) and Open (blue background) motor domains presented in this work. "LIS1" indicates how many LIS1 β-propellers are bound to the dynein motor. "Linker" refers to the overall conformation (bent, intermediate, straight) of that domain. "Stalk" refers to the register between the two helices in the coiled-coil [4]. The nucleotide states of AAA1-AAA4 are based on our interpretation of the densities in our maps.

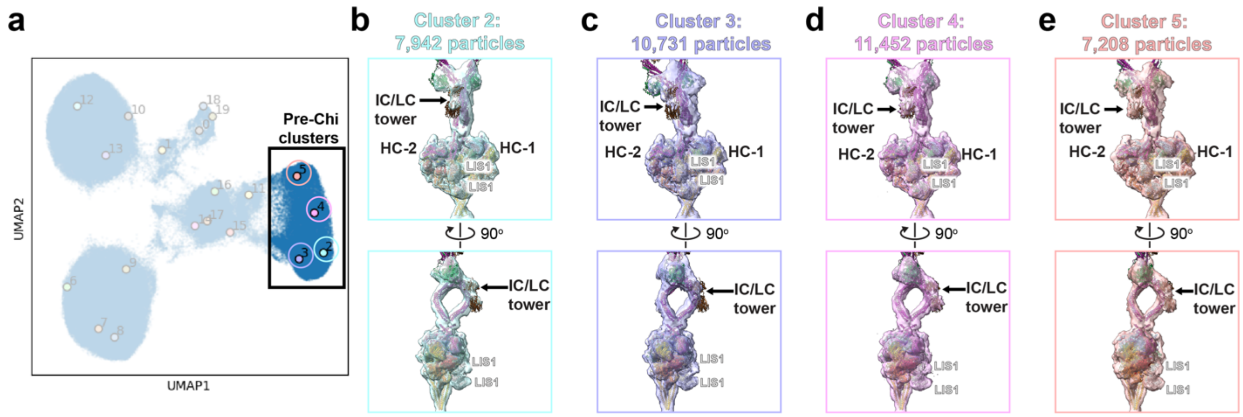

Supplementary Figure 8. LIS1 binds preferentially to one face on the Pre-Chi structure.

a. cryoDRGN UMAP representation. This analysis focuses on the Pre-Chi k means clusters 2-5 (highlighted by the box) from Supplementary Figure 3. **b-e.** Volumes from clusters 2-5 shown in two views: front view of the IC/LC tower face (top row) and side view (bottom row). The IC-LC tower (labeled) is used as a reference to determine which side of Phi LIS1 is bound to.

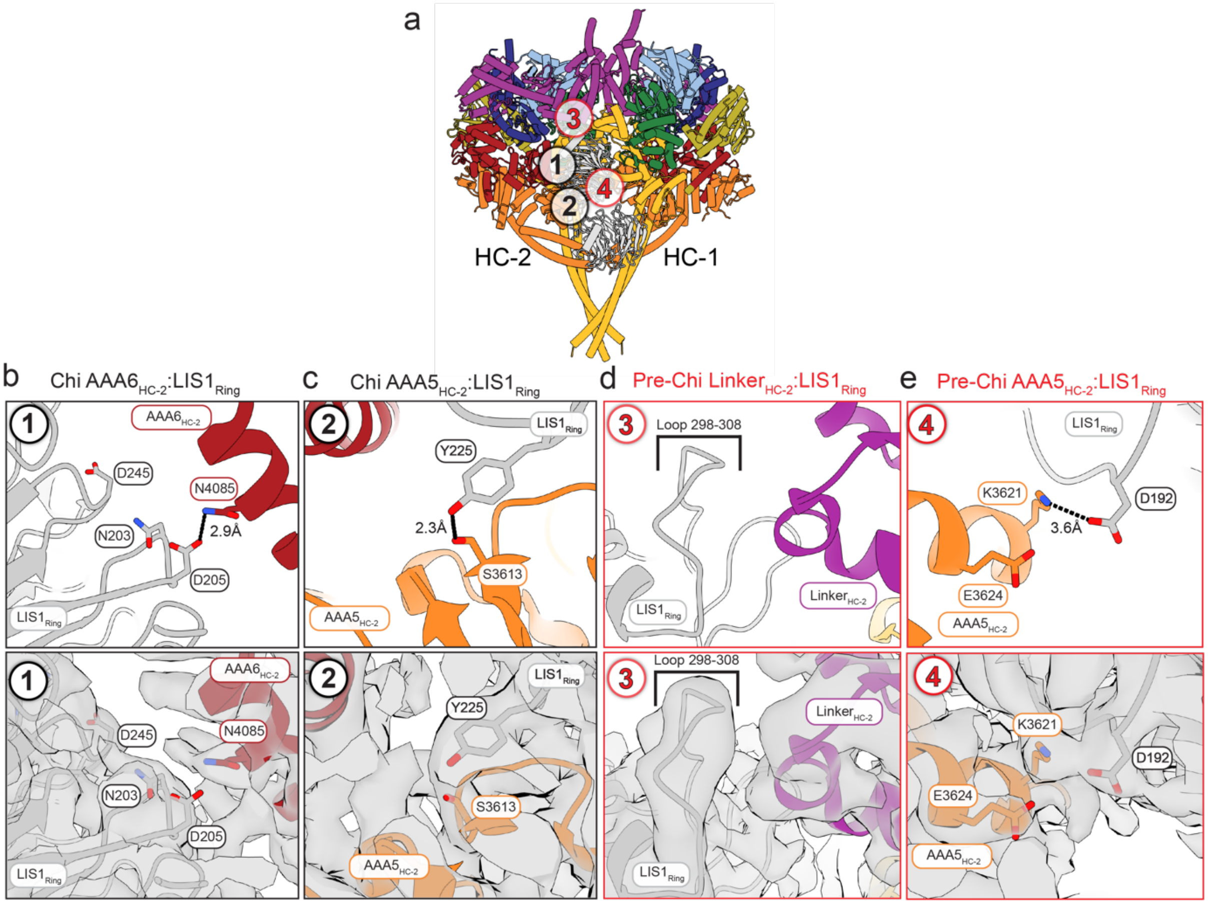

Supplementary Figure 9. Volume Overlay of Pre-Chi Interfaces.

a. Pre-Chi model viewed from the LIS1-bound face with interfaces present in both Chi and Pre-Chi (#1-2 in black circles) and Pre-Chi-specific interfaces (#3-4 in red circles) highlighted. **b-e.** Close ups of the interfaces highlighted in (a). The top row is the atomic model of Pre-Chi, and the bottom row is the locally filtered map of Pre-Chi superimposed onto the atomic model of Pre-Chi. Residues involved in the interfaces and the names of the domains interacting with $LIS1_{ring}$ are highlighted in each panel. Interactions are shown with dotted lines, with their distances (in Å) indicated.

**Description**										
Sample	Full-length Human Cytoplasmic Dynein-1 + Human LIS1 in the presence of 1mM ATP									
Class Name	Pre-Chi Motor	Pre-Chi Tail	Phi Motor	Phi Tail	Bent /2xLIS1	Bent	Bent. /1xLIS1	Interm.	Straight /1xLIS1	Straight
EMD-	47342	47430	47373	47443	47370	47360	47372	47371	47429	47377
PDB-	9DZY	9E23	9E0X	9E28	9E0T	9E0K	9E0W	9E0U	9E22	9E0Y
**Data collection and processing**										
Facility	UC San Diego Cryo-EM facility									
Microscope	Titan Krios G4									
Voltage(kV)	300									
Camera	Falcon 4									
Magnification	130,000									
Electron exposure (e/Å^2)	55									
Defocus range (μm)	0.6-3.3									
Pixel size (Å)	0.935									
Symmetry imposed	C1									
Images (no.)	11,686									
Initial particles (no.)	1,937,842									
Final particles (no.)	66880	12517	161043	21400	23289	22570	26127	13958	69831	50952
Map resolution (Å) (FSC 0.143)	3.1	6.2	2.7	4.4	3.1	3.2	3.2	3.4	3.3	3.4
**Refinement**										
Initial model used (PDB code)	5NUG, 8DYU	8PTK	5NUG	8PTK	8DYU	8DYU	8DYU	8DYU	8PQV, 8DYU	8PQV
Model resolution (Å) (FSC 0.5)	3.3	7.5	2.9	6.5	3.5	3.6	3.6	3.8	3.8	4.3

Map sharpening *B* factor (Å^2)	50.3	342	67.4	47.3	35.8	29.1	37	17	47.5	37.5
**Model composition**										
Non-hydrogen atoms	51122	21249	46234	21249	27918	22930	24531	22051	24612	22201
Protein residues	6466	4734	5840	4734	3518	2892	3095	2783	3245	2753
Ligands	MG:4, ADP:6, ATP:2	-	MG:4, ADP:6, ATP:2	-	MG:2, ADP:3, ATP:1	MG:2, ADP:3, ATP:1	MG:2, ADP:3, ATP:1	MG:2, ADP:3, ATP:1	MG:1, ADP:2, ATP:2	ADP:2, ATP:2
***B* factors (Å^2)**										
Protein	123.0	564.5	71.5	445.1	164.1	104.9	114.0	130.8	154.9	207.5
Ligand	94.38	-	37.7	-	115.4	72.7	66.1	84.1	85.3	141.7
**R.m.s. deviations**										
Bond lengths (Å)	0.004	0.002	0.003	0.002	0.003	0.003	0.003	0.004	0.003	0.004
Bond angles (°)	0.651	0.484	0.640	0.481	0.644	0.644	0.611	0.673	0.627	0.764
**Validation**										
MolProbity score	1.57	1.93	1.37	1.68	1.57	1.56	1.58	1.53	1.45	1.74
Clash score	6.05	8.75	4.60	4.24	6.46	5.81	6.21	5.65	5.09	7.49
Rotamer outliers (%)	0	0	0.04	0	0.03	0.04	0.04	0	0	0.04
**Ramachandran plot**										
Outliers (%)	0.02	0.13	0.03	0.11	0	0	0	0	0	0
Allowed (%)	3.58	7.14	2.71	7.48	3.39	3.60	3.62	3.45	3.06	4.77
Favored (%)	96.41	92.73	97.25	92.41	96.61	96.40	96.38	96.55	96.91	95.23

Supplementary Table: 1 Cryo-EM data and model validation statistics.

Cryo-EM data collection parameters, reconstruction, and model refinement statistics.
