# Supplementary material for: Cryo-EM captures early intermediate steps in dynein activation by LIS1
Source: Nat Commun. 2025 Aug 1;16:7054. doi: 10.1038/s41467-025-62185-z (PMC12317079; doi:10.1038/s41467-025-62185-z)
Supplement: Supplementary file 2 — Description of Additional Supplementary Files [file 41467_2025_62185_MOESM2_ESM.pdf]

Description of Additional Supplementary Files

File name: Supplementary Movie 1
Description: The movie illustrates a morph between eight distinct dynein-LIS1 and dynein motor structures, showing transitions in both the cryo-EM density maps and corresponding atomic models.

File name: Supplementary Movie 2
Description: The movie illustrates the full-length Pre-Chi and Phi models, highlighting structural changes and α carbon displacements between the two conformations.

File name: Supplementary Movie 3
Description: The movie depicts structural changes between the Phi and Pre-Chi models, showing how Phi stabilizing interfaces becomes disrupted in Pre-Chi.

File name: Supplementary Movie 4
Description: The movie illustrates the Pre-Chi and Chi models, highlighting structural changes and α carbon displacements between the two conformations.

File name: Supplementary Movie 5
Description: The movie illustrates the Pre-Chi model, highlighting Chi shared interfaces as well as interfaces that is specific to Pre-Chi.
